# 4-[Bis(4-fluorophenyl)methyl]piperazin-1-ium picrate

**DOI:** 10.1107/S160053681103580X

**Published:** 2011-09-14

**Authors:** Richard Betz, Thomas Gerber, Eric Hosten, Alaloor S. Dayananda, Hemmige S. Yathirajan, Badiadka Narayana

**Affiliations:** a Nelson Mandela Metropolitan University, Summerstrand Campus, Department of Chemistry, University Way, Summerstrand, PO Box 77000, Port Elizabeth, 6031, South Africa; b University of Mysore, Department of Studies in Chemistry, Manasagangotri, Mysore 570 006, India; c Mangalore University, Department of Studies in Chemistry, Mangalagangotri 574 199, India

## Abstract

The title compound {systematic name: 4-[bis(4-fluorophenyl)methyl]piperazin-1-ium 2,4,6-tri­nitro­phenolate}, C_17_H_19_F_2_N_2_^+^·C_6_H_2_N_3_O_7_^−^,  is the picrate salt of a piperazine-supported amine bearing a benzhydryl substituent on one of its N atoms. During co-crystallisation, protonation took place on the N atom of the secondary amine functionality. The non-aromatic six-membered heterocycle adopts a chair conformation. In the crystal, N—H⋯O hydrogen bonds as well as C—H⋯O contacts connect the components into a three-dimensional network.

## Related literature

For background to the biological activity of piperazines, see: Brockunier *et al.* (2004 ▶); Bogatcheva *et al.* (2006 ▶). For related structures, see: Jasinski *et al.* (2010 ▶, 2011 ▶); Dutkiewicz *et al.* (2011 ▶). For graph-set analysis of hydrogen bonds, see: Etter *et al.* (1990 ▶); Bernstein *et al.* (1995 ▶). For puckering analysis, see: Cremer & Pople (1975 ▶).
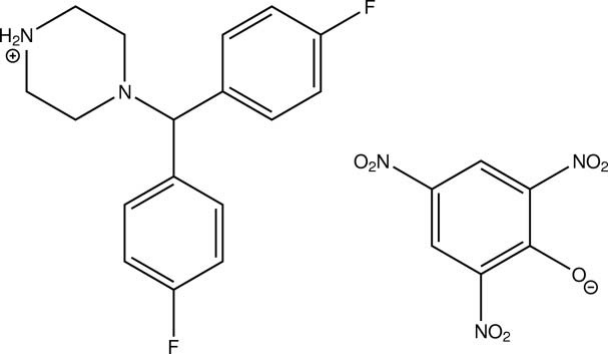


## Experimental

### 

#### Crystal data

C_17_H_19_F_2_N_2_^+^·C_6_H_2_N_3_O_7_^−^
*M* *_r_* = 517.45
Monoclinic, 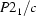

*a* = 8.9425 (2) Å
*b* = 11.8286 (2) Å
*c* = 23.0922 (4) Å
β = 105.720 (1)°
*V* = 2351.27 (8) Å^3^
*Z* = 4
Mo *K*α radiation
μ = 0.12 mm^−1^
*T* = 200 K
0.52 × 0.49 × 0.41 mm

#### Data collection

Bruker APEXII CCD diffractometer
Absorption correction: multi-scan (*SADABS*: Bruker, 2008 ▶) *T*_min_ = 0.928, *T*_max_ = 1.000
20823 measured reflections
5842 independent reflections
5041 reflections with *I* > 2σ(*I*)
*R*_int_ = 0.013

#### Refinement

*R*[*F* ^2^ > 2σ(*F* ^2^)] = 0.039
*wR*(*F* ^2^) = 0.111
*S* = 1.03
5842 reflections
342 parameters
H atoms treated by a mixture of independent and constrained refinement
Δρ_max_ = 0.32 e Å^−3^
Δρ_min_ = −0.31 e Å^−3^

### 

Data collection: *APEX2* (Bruker, 2010 ▶); cell refinement: *SAINT* (Bruker, 2010 ▶); data reduction: *SAINT*; program(s) used to solve structure: *SHELXS97* (Sheldrick, 2008 ▶); program(s) used to refine structure: *SHELXL97* (Sheldrick, 2008 ▶); molecular graphics: *ORTEPIII* (Farrugia, 1997 ▶) and *Mercury* (Macrae *et al.*, 2008 ▶); software used to prepare material for publication: *SHELXL97* and *PLATON* (Spek, 2009 ▶).

## Supplementary Material

Crystal structure: contains datablock(s) I, global. DOI: 10.1107/S160053681103580X/hg5090sup1.cif

Supplementary material file. DOI: 10.1107/S160053681103580X/hg5090Isup2.cdx

Structure factors: contains datablock(s) I. DOI: 10.1107/S160053681103580X/hg5090Isup3.hkl

Supplementary material file. DOI: 10.1107/S160053681103580X/hg5090Isup4.cml

Additional supplementary materials:  crystallographic information; 3D view; checkCIF report

## Figures and Tables

**Table 1:** Hydrogen-bond geometry (Å, °)

*D*—H⋯*A*	*D*—H	H⋯*A*	*D*⋯*A*	*D*—H⋯*A*
N2—H721⋯O1^i^	0.909 (17)	1.924 (17)	2.7696 (12)	154.0 (14)
N2—H721⋯O52^i^	0.909 (17)	2.369 (16)	3.0018 (14)	126.7 (12)
N2—H722⋯O1^ii^	0.926 (16)	1.927 (16)	2.7402 (13)	145.4 (14)
N2—H722⋯O31^ii^	0.926 (16)	2.345 (16)	3.0791 (15)	136.0 (13)
C3—H3*A*⋯O52^i^	0.99	2.60	3.0002 (16)	104
C13—H13⋯O32^iii^	0.95	2.39	3.2159 (16)	145
C23—H23⋯O42^iv^	0.95	2.61	3.2827 (17)	129

Symmetry codes: (i) 
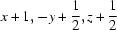
; (ii) 
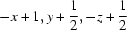
; (iii) 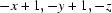; (iv) 
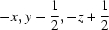
.

**Fig. 1.**
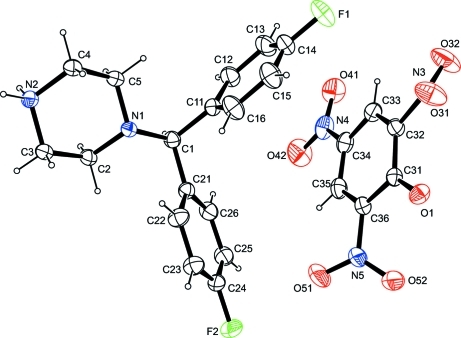
The molecular structure of the title compound, with atom labels and anisotropic displacement ellipsoids (drawn at 50% probability level).

**Fig. 2.**
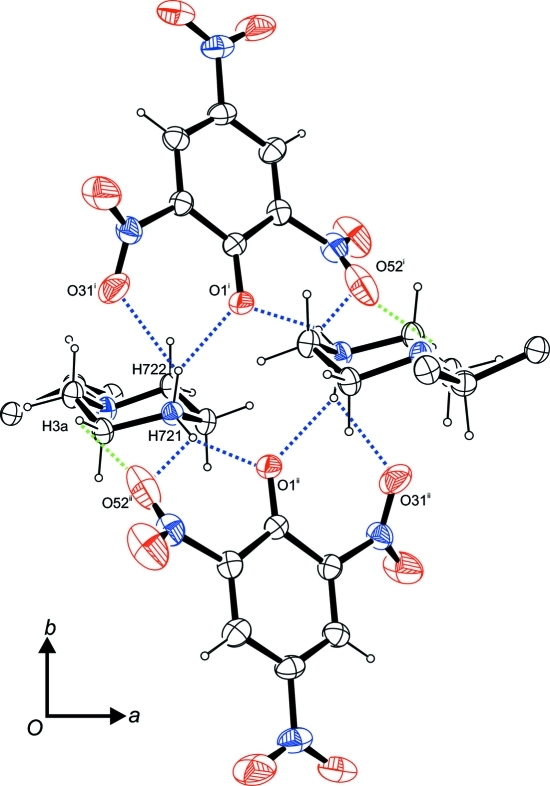
Selected intermolecular contacts, viewed along [0 0 - 1]. For clarity, the aromatic substituents on the tertiary nitrogen atom are omitted. Green dashed lines indicate C–H···O contacts, blue dashed lines indicate classical hydrogen bonds. Symmetry operators: i -x + 1, y + 1/2, -z + 1/2; iix + 1, -y + 1/2, z + 1/2.

**Fig. 3.**
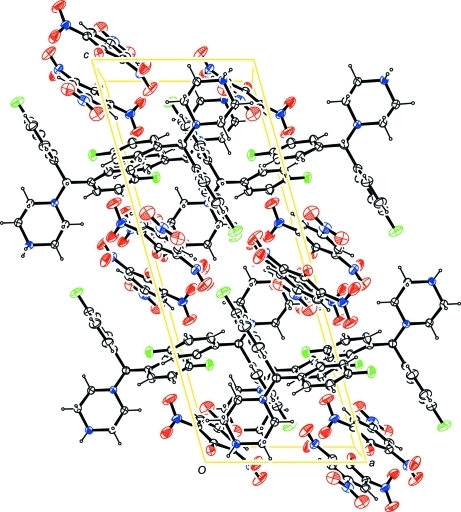
Molecular packing of the title compound, viewed along [0 1 0] (anisotropic displacement ellipsoids drawn at 50% probability level).

## supplementary crystallographic information

### Comment

4,4'-Difluorobenzhydryl piperazine is an intermediate for the preparation of flunarizine which is a calcium channel blocker. Piperazines are among the most important building blocks in today's drug discovery. They are found in biologically active compounds across a number of different therapeutic areas such as antifungal, antibacterial, antimalarial, antipsychotic, antidepressant and antitumour activity against colon, prostate, breast, lung and leukemia tumors (Brockunier *et al.*, 2004; Bogatcheva *et al.*, 2006). The crystal structures of some related salts *viz*., levocetirizinium dipicrate (Jasinski *et al.*, 2010), cinnarizinium dipicrate (Jasinski *et al.*, 2011), 1-methylpiperazine-1,4-diium dipicrate (Dutkiewicz *et al.*, 2011), have been reported. In the course of our studies on picrates of simple organic cations and in view of the importance of piperazines, we have determined the crystal and molecular structure of the title salt.

Protonation occurred at the nitrogen atom that is part of the secondary amine functionality. The non-aromatic heterocycle adopts a *chair* conformation (^1^*C*_4_, ^N1^*C*_N2_) according to a conformation analysis (Cremer & Pople, 1975). The least-squares planes defined by the carbon atoms of the two fluorophenyl moieties intersect at an angle of 73.71 (4) °. The planes defined by the atoms of the nitro groups on the picrate anion enclose angles of 7.62 (14) °, 33.52 (18) ° and 37.08 (17) °, respectively with the aromatic system they are bonded to (Fig. 1).

In the crystal, hydrogen bonds of the N–H···O type as well as C–H···O contacts are present. Both classical hydrogen bonds show bifurcation between the phenolic O atom as well as one of the nitro group's O atoms. The C–H···O contacts are supported by one of the hydrogen atoms of the non-aromatic heterocycle as well as one of the hydrogen atoms in *ortho* position to F atom in each of the fluorophenyl moieties. Apart from one O atom of a nitro group that forms a N–O···*C*_g_ contact with a neighbouring picrate anion, all O atoms act as acceptors for hydrogen atoms. In terms of graph-set analysis (Etter *et al.*, 1990; Bernstein *et al.*, 1995), the descriptor for the classical hydrogen bonds is *DDDD* on the unitary level while the C–H···O contacts require a *DD* descriptor on the same level. In total, the entities of the title compound are connected to a three-dimensional network in the crystal. The shortest intercentroid distance between two centers of gravity was found at 4.0808 (7) Å and is apparent between one of the fluorophenyl moieties and the picrate anion (Fig. 2).

The packing of the title compound in the crystal structure is shown in Figure 3.

### Experimental

4,4'-Difluorobenzhydryl piperazine was obtained from *R. L*. Fine Chem., Bengaluru, India. 4,4'-Difluorobenzhydryl piperazine (2.88 g, 0.01 mol) was dissolved in 10 ml of methanol and picric acid (2.29 g, 0.01 mol) was also dissolved in 10 ml of methanol. Both the solutions were mixed and stirred in a beaker at 333 K for 30 minutes. The mixture was kept aside for a day at room temperature. The salt formed was filtered & dried in vaccum desiccator over phosphorous pentoxide. The compound was recrystallized from a mixture of (1:1) acetone and acetonitrile by slow evaporation (m.p: 513–516 K).

### Refinement

Carbon-bound H atoms were placed in calculated positions (C—H 0.95 Å for aromatic carbon atoms, C—H 0.99 Å for methylene groups) and were included in the refinement in the riding model approximation, with *U*(H) set to 1.2*U*_eq_(C). Both nitrogen-bound H atoms were located on a difference Fourier map and refined freely.

### Crystal data

**Table:**

C_17_H_19_F_2_N_2_^+^·C_6_H_2_N_3_O_7_^−^	*F*(000) = 1072
*M**_r_* = 517.45	*D*_x_ = 1.462 Mg m^−^^3^
Monoclinic, *P*2_1_/*c*	Mo *K*α radiation, λ = 0.71073 Å
Hall symbol: -P 2ybc	Cell parameters from 9984 reflections
*a* = 8.9425 (2) Å	θ = 2.5–28.3°
*b* = 11.8286 (2) Å	µ = 0.12 mm^−^^1^
*c* = 23.0922 (4) Å	*T* = 200 K
β = 105.720 (1)°	Block, yellow
*V* = 2351.27 (8) Å^3^	0.52 × 0.49 × 0.41 mm
*Z* = 4	

### Data collection

**Table:**

Bruker APEXII CCD diffractometer	5842 independent reflections
Radiation source: fine-focus sealed tube	5041 reflections with *I* > 2σ(*I*)
graphite	*R*_int_ = 0.013
φ and ω scans	θ_max_ = 28.3°, θ_min_ = 2.0°
Absorption correction: multi-scan (*SADABS*: Bruker, 2008)	*h* = −10→11
*T*_min_ = 0.928, *T*_max_ = 1.000	*k* = −15→13
20823 measured reflections	*l* = −30→30

### Refinement

**Table:**

Refinement on *F*^2^	Primary atom site location: structure-invariant direct methods
Least-squares matrix: full	Secondary atom site location: difference Fourier map
*R*[*F*^2^ > 2σ(*F*^2^)] = 0.039	Hydrogen site location: inferred from neighbouring sites
*wR*(*F*^2^) = 0.111	H atoms treated by a mixture of independent and constrained refinement
*S* = 1.03	*w* = 1/[σ^2^(*F*_o_^2^) + (0.058*P*)^2^ + 0.8015*P*] where *P* = (*F*_o_^2^ + 2*F*_c_^2^)/3
5842 reflections	(Δ/σ)_max_ < 0.001
342 parameters	Δρ_max_ = 0.32 e Å^−^^3^
0 restraints	Δρ_min_ = −0.31 e Å^−^^3^

### Fractional atomic coordinates and isotropic or equivalent isotropic displacement parameters (Å^2^)

**Table:**

	*x*	*y*	*z*	*U*_iso_*/*U*_eq_	
F1	0.59114 (13)	0.42083 (9)	0.07691 (4)	0.0571 (3)	
F2	−0.17242 (8)	0.30044 (8)	0.27076 (4)	0.0414 (2)	
N1	0.53229 (10)	0.45389 (8)	0.34157 (4)	0.02357 (19)	
N2	0.77494 (11)	0.44880 (8)	0.45221 (4)	0.02322 (19)	
H721	0.8435 (19)	0.4073 (14)	0.4805 (7)	0.036 (4)*	
H722	0.7972 (18)	0.5235 (14)	0.4633 (7)	0.035 (4)*	
C1	0.41152 (13)	0.49894 (9)	0.28976 (5)	0.0243 (2)	
H1	0.4030	0.5824	0.2952	0.029*	
C2	0.49947 (13)	0.48107 (10)	0.39896 (5)	0.0256 (2)	
H2A	0.3914	0.4593	0.3972	0.031*	
H2B	0.5104	0.5635	0.4064	0.031*	
C3	0.61166 (12)	0.41795 (10)	0.44964 (5)	0.0252 (2)	
H3A	0.5902	0.4370	0.4884	0.030*	
H3B	0.5970	0.3355	0.4431	0.030*	
C4	0.80775 (12)	0.43290 (10)	0.39261 (5)	0.0259 (2)	
H4A	0.8047	0.3514	0.3826	0.031*	
H4B	0.9129	0.4618	0.3945	0.031*	
C5	0.68813 (12)	0.49562 (10)	0.34431 (5)	0.0254 (2)	
H5A	0.6942	0.5776	0.3534	0.030*	
H5B	0.7098	0.4844	0.3049	0.030*	
C11	0.45589 (13)	0.47782 (10)	0.23136 (5)	0.0255 (2)	
C12	0.48205 (16)	0.56737 (11)	0.19670 (6)	0.0357 (3)	
H12	0.4681	0.6426	0.2087	0.043*	
C13	0.52840 (18)	0.54931 (12)	0.14453 (6)	0.0423 (3)	
H13	0.5465	0.6111	0.1210	0.051*	
C14	0.54715 (16)	0.44020 (13)	0.12803 (5)	0.0369 (3)	
C15	0.51902 (18)	0.34899 (12)	0.16014 (6)	0.0417 (3)	
H15	0.5313	0.2741	0.1473	0.050*	
C16	0.47223 (17)	0.36882 (11)	0.21181 (6)	0.0372 (3)	
H16	0.4509	0.3065	0.2343	0.045*	
C21	0.25510 (12)	0.44523 (9)	0.28664 (5)	0.0241 (2)	
C22	0.24318 (14)	0.33552 (10)	0.30656 (6)	0.0303 (2)	
H22	0.3351	0.2936	0.3238	0.036*	
C23	0.09940 (14)	0.28616 (11)	0.30173 (6)	0.0321 (3)	
H23	0.0917	0.2115	0.3158	0.039*	
C24	−0.03126 (13)	0.34853 (11)	0.27601 (5)	0.0304 (2)	
C25	−0.02518 (14)	0.45697 (12)	0.25579 (6)	0.0335 (3)	
H25	−0.1178	0.4978	0.2381	0.040*	
C26	0.11954 (14)	0.50573 (11)	0.26174 (5)	0.0298 (2)	
H26	0.1260	0.5813	0.2487	0.036*	
O1	0.04838 (9)	0.14124 (7)	0.02412 (4)	0.02909 (18)	
O31	0.31391 (13)	0.16472 (9)	−0.00959 (5)	0.0496 (3)	
O32	0.30392 (17)	0.32242 (10)	−0.05643 (6)	0.0588 (3)	
O41	0.22278 (14)	0.65139 (8)	0.05559 (5)	0.0485 (3)	
O42	0.08126 (16)	0.63785 (9)	0.11771 (6)	0.0578 (3)	
O51	−0.12687 (16)	0.26580 (12)	0.15001 (5)	0.0641 (4)	
O52	−0.20063 (15)	0.17435 (12)	0.06807 (5)	0.0598 (4)	
N3	0.27271 (13)	0.26325 (9)	−0.01814 (5)	0.0323 (2)	
N4	0.14537 (14)	0.59644 (9)	0.08192 (5)	0.0380 (3)	
N5	−0.11622 (13)	0.24272 (10)	0.10035 (5)	0.0349 (2)	
C31	0.07480 (12)	0.24316 (9)	0.03909 (5)	0.0233 (2)	
C32	0.18288 (13)	0.31337 (10)	0.01940 (5)	0.0258 (2)	
C33	0.20463 (14)	0.42683 (10)	0.03198 (5)	0.0284 (2)	
H33	0.2723	0.4702	0.0154	0.034*	
C34	0.12583 (14)	0.47641 (10)	0.06937 (5)	0.0299 (2)	
C35	0.02432 (14)	0.41477 (10)	0.09322 (5)	0.0307 (2)	
H35	−0.0260	0.4491	0.1201	0.037*	
C36	−0.00186 (13)	0.30330 (10)	0.07723 (5)	0.0270 (2)	

### Atomic displacement parameters (Å^2^)

**Table:**

	*U*^11^	*U*^22^	*U*^33^	*U*^12^	*U*^13^	*U*^23^
F1	0.0726 (6)	0.0721 (7)	0.0364 (4)	0.0016 (5)	0.0315 (4)	−0.0020 (4)
F2	0.0245 (4)	0.0574 (5)	0.0434 (4)	−0.0087 (3)	0.0112 (3)	−0.0072 (4)
N1	0.0199 (4)	0.0306 (5)	0.0207 (4)	−0.0002 (3)	0.0064 (3)	0.0021 (3)
N2	0.0215 (4)	0.0250 (5)	0.0227 (4)	0.0005 (3)	0.0052 (3)	0.0024 (3)
C1	0.0248 (5)	0.0242 (5)	0.0238 (5)	0.0009 (4)	0.0064 (4)	0.0029 (4)
C2	0.0218 (5)	0.0334 (6)	0.0230 (5)	0.0022 (4)	0.0081 (4)	0.0020 (4)
C3	0.0226 (5)	0.0306 (5)	0.0238 (5)	−0.0013 (4)	0.0086 (4)	0.0034 (4)
C4	0.0219 (5)	0.0315 (6)	0.0258 (5)	0.0015 (4)	0.0090 (4)	−0.0003 (4)
C5	0.0224 (5)	0.0311 (6)	0.0241 (5)	−0.0019 (4)	0.0089 (4)	0.0024 (4)
C11	0.0241 (5)	0.0297 (5)	0.0219 (5)	−0.0011 (4)	0.0045 (4)	0.0023 (4)
C12	0.0472 (7)	0.0301 (6)	0.0318 (6)	−0.0115 (5)	0.0144 (5)	−0.0011 (5)
C13	0.0555 (8)	0.0427 (7)	0.0331 (6)	−0.0150 (6)	0.0195 (6)	0.0033 (5)
C14	0.0366 (6)	0.0519 (8)	0.0244 (5)	−0.0009 (6)	0.0118 (5)	−0.0008 (5)
C15	0.0556 (8)	0.0374 (7)	0.0349 (7)	0.0105 (6)	0.0171 (6)	0.0008 (5)
C16	0.0530 (8)	0.0298 (6)	0.0321 (6)	0.0081 (6)	0.0173 (6)	0.0077 (5)
C21	0.0227 (5)	0.0270 (5)	0.0222 (5)	0.0013 (4)	0.0054 (4)	0.0008 (4)
C22	0.0242 (5)	0.0278 (6)	0.0371 (6)	0.0017 (4)	0.0053 (4)	0.0045 (5)
C23	0.0303 (6)	0.0304 (6)	0.0359 (6)	−0.0034 (5)	0.0091 (5)	0.0006 (5)
C24	0.0230 (5)	0.0428 (7)	0.0267 (5)	−0.0046 (5)	0.0091 (4)	−0.0067 (5)
C25	0.0243 (5)	0.0453 (7)	0.0308 (6)	0.0085 (5)	0.0076 (4)	0.0042 (5)
C26	0.0292 (6)	0.0319 (6)	0.0292 (5)	0.0064 (5)	0.0094 (4)	0.0058 (4)
O1	0.0264 (4)	0.0238 (4)	0.0361 (4)	−0.0003 (3)	0.0067 (3)	−0.0050 (3)
O31	0.0572 (7)	0.0395 (5)	0.0658 (7)	0.0152 (5)	0.0400 (6)	0.0056 (5)
O32	0.0885 (9)	0.0463 (6)	0.0618 (7)	−0.0101 (6)	0.0547 (7)	0.0002 (5)
O41	0.0624 (7)	0.0266 (5)	0.0541 (6)	−0.0054 (4)	0.0115 (5)	0.0024 (4)
O42	0.0807 (9)	0.0339 (5)	0.0635 (7)	0.0032 (5)	0.0279 (6)	−0.0179 (5)
O51	0.0808 (9)	0.0832 (9)	0.0408 (6)	−0.0316 (7)	0.0379 (6)	−0.0244 (6)
O52	0.0595 (7)	0.0789 (8)	0.0511 (6)	−0.0385 (6)	0.0324 (5)	−0.0295 (6)
N3	0.0350 (5)	0.0328 (5)	0.0340 (5)	−0.0041 (4)	0.0176 (4)	−0.0032 (4)
N4	0.0454 (6)	0.0256 (5)	0.0375 (6)	0.0030 (4)	0.0016 (5)	−0.0041 (4)
N5	0.0383 (6)	0.0413 (6)	0.0293 (5)	−0.0061 (5)	0.0162 (4)	−0.0088 (4)
C31	0.0234 (5)	0.0244 (5)	0.0208 (5)	0.0014 (4)	0.0038 (4)	−0.0010 (4)
C32	0.0263 (5)	0.0277 (5)	0.0239 (5)	0.0007 (4)	0.0078 (4)	−0.0014 (4)
C33	0.0295 (5)	0.0268 (5)	0.0272 (5)	−0.0020 (4)	0.0045 (4)	0.0011 (4)
C34	0.0345 (6)	0.0227 (5)	0.0287 (5)	0.0015 (4)	0.0019 (4)	−0.0036 (4)
C35	0.0335 (6)	0.0321 (6)	0.0255 (5)	0.0033 (5)	0.0064 (4)	−0.0067 (4)
C36	0.0277 (5)	0.0315 (6)	0.0223 (5)	−0.0015 (4)	0.0078 (4)	−0.0036 (4)

### Geometric parameters (Å, °)

**Table:**

F1—C14	1.3610 (14)	C15—H15	0.9500
F2—C24	1.3597 (13)	C16—H16	0.9500
N1—C5	1.4636 (13)	C21—C22	1.3905 (16)
N1—C2	1.4686 (13)	C21—C26	1.3909 (15)
N1—C1	1.4770 (14)	C22—C23	1.3888 (17)
N2—C3	1.4906 (14)	C22—H22	0.9500
N2—C4	1.4947 (14)	C23—C24	1.3734 (18)
N2—H721	0.909 (17)	C23—H23	0.9500
N2—H722	0.926 (16)	C24—C25	1.3711 (19)
C1—C21	1.5202 (15)	C25—C26	1.3893 (18)
C1—C11	1.5258 (15)	C25—H25	0.9500
C1—H1	1.0000	C26—H26	0.9500
C2—C3	1.5158 (15)	O1—C31	1.2587 (13)
C2—H2A	0.9900	O31—N3	1.2221 (15)
C2—H2B	0.9900	O32—N3	1.2179 (14)
C3—H3A	0.9900	O41—N4	1.2245 (16)
C3—H3B	0.9900	O42—N4	1.2282 (16)
C4—C5	1.5140 (15)	O51—N5	1.2073 (14)
C4—H4A	0.9900	O52—N5	1.2143 (15)
C4—H4B	0.9900	N3—C32	1.4580 (14)
C5—H5A	0.9900	N4—C34	1.4501 (15)
C5—H5B	0.9900	N5—C36	1.4630 (15)
C11—C12	1.3854 (16)	C31—C32	1.4383 (15)
C11—C16	1.3869 (17)	C31—C36	1.4418 (15)
C12—C13	1.3926 (18)	C32—C33	1.3757 (16)
C12—H12	0.9500	C33—C34	1.3841 (17)
C13—C14	1.369 (2)	C33—H33	0.9500
C13—H13	0.9500	C34—C35	1.3891 (18)
C14—C15	1.370 (2)	C35—C36	1.3721 (17)
C15—C16	1.3870 (18)	C35—H35	0.9500
			
C5—N1—C2	107.93 (8)	C14—C15—C16	118.33 (13)
C5—N1—C1	113.08 (8)	C14—C15—H15	120.8
C2—N1—C1	111.77 (8)	C16—C15—H15	120.8
C3—N2—C4	111.54 (8)	C11—C16—C15	121.35 (12)
C3—N2—H721	111.0 (10)	C11—C16—H16	119.3
C4—N2—H721	109.4 (10)	C15—C16—H16	119.3
C3—N2—H722	112.3 (10)	C22—C21—C26	118.73 (11)
C4—N2—H722	107.0 (10)	C22—C21—C1	121.76 (10)
H721—N2—H722	105.3 (14)	C26—C21—C1	119.49 (10)
N1—C1—C21	110.43 (8)	C23—C22—C21	121.20 (11)
N1—C1—C11	110.40 (9)	C23—C22—H22	119.4
C21—C1—C11	110.24 (9)	C21—C22—H22	119.4
N1—C1—H1	108.6	C24—C23—C22	118.01 (12)
C21—C1—H1	108.6	C24—C23—H23	121.0
C11—C1—H1	108.6	C22—C23—H23	121.0
N1—C2—C3	109.67 (9)	F2—C24—C25	118.85 (11)
N1—C2—H2A	109.7	F2—C24—C23	118.32 (12)
C3—C2—H2A	109.7	C25—C24—C23	122.84 (11)
N1—C2—H2B	109.7	C24—C25—C26	118.46 (11)
C3—C2—H2B	109.7	C24—C25—H25	120.8
H2A—C2—H2B	108.2	C26—C25—H25	120.8
N2—C3—C2	110.27 (9)	C25—C26—C21	120.76 (11)
N2—C3—H3A	109.6	C25—C26—H26	119.6
C2—C3—H3A	109.6	C21—C26—H26	119.6
N2—C3—H3B	109.6	O32—N3—O31	123.16 (11)
C2—C3—H3B	109.6	O32—N3—C32	118.00 (11)
H3A—C3—H3B	108.1	O31—N3—C32	118.83 (10)
N2—C4—C5	109.93 (9)	O41—N4—O42	123.52 (12)
N2—C4—H4A	109.7	O41—N4—C34	118.30 (11)
C5—C4—H4A	109.7	O42—N4—C34	118.16 (12)
N2—C4—H4B	109.7	O51—N5—O52	122.53 (12)
C5—C4—H4B	109.7	O51—N5—C36	118.60 (11)
H4A—C4—H4B	108.2	O52—N5—C36	118.81 (10)
N1—C5—C4	109.94 (9)	O1—C31—C32	123.95 (10)
N1—C5—H5A	109.7	O1—C31—C36	123.74 (10)
C4—C5—H5A	109.7	C32—C31—C36	112.31 (10)
N1—C5—H5B	109.7	C33—C32—C31	124.42 (10)
C4—C5—H5B	109.7	C33—C32—N3	116.94 (10)
H5A—C5—H5B	108.2	C31—C32—N3	118.60 (10)
C12—C11—C16	118.26 (11)	C32—C33—C34	118.57 (11)
C12—C11—C1	120.70 (11)	C32—C33—H33	120.7
C16—C11—C1	121.04 (10)	C34—C33—H33	120.7
C11—C12—C13	121.27 (12)	C33—C34—C35	121.51 (11)
C11—C12—H12	119.4	C33—C34—N4	119.03 (11)
C13—C12—H12	119.4	C35—C34—N4	119.41 (11)
C14—C13—C12	118.23 (12)	C36—C35—C34	118.68 (11)
C14—C13—H13	120.9	C36—C35—H35	120.7
C12—C13—H13	120.9	C34—C35—H35	120.7
F1—C14—C13	119.08 (12)	C35—C36—C31	124.32 (11)
F1—C14—C15	118.38 (13)	C35—C36—N5	117.23 (10)
C13—C14—C15	122.51 (12)	C31—C36—N5	118.45 (10)
			
C5—N1—C1—C21	175.43 (9)	C22—C23—C24—C25	−0.79 (19)
C2—N1—C1—C21	−62.52 (11)	F2—C24—C25—C26	179.53 (11)
C5—N1—C1—C11	53.26 (12)	C23—C24—C25—C26	−0.13 (18)
C2—N1—C1—C11	175.31 (9)	C24—C25—C26—C21	1.15 (18)
C5—N1—C2—C3	−63.90 (11)	C22—C21—C26—C25	−1.20 (17)
C1—N1—C2—C3	171.15 (9)	C1—C21—C26—C25	177.04 (11)
C4—N2—C3—C2	−53.24 (12)	O1—C31—C32—C33	175.18 (11)
N1—C2—C3—N2	58.60 (12)	C36—C31—C32—C33	−3.91 (16)
C3—N2—C4—C5	53.23 (12)	O1—C31—C32—N3	−2.65 (17)
C2—N1—C5—C4	64.26 (11)	C36—C31—C32—N3	178.26 (10)
C1—N1—C5—C4	−171.57 (9)	O32—N3—C32—C33	−32.04 (17)
N2—C4—C5—N1	−58.93 (12)	O31—N3—C32—C33	147.48 (12)
N1—C1—C11—C12	−116.94 (12)	O32—N3—C32—C31	145.96 (12)
C21—C1—C11—C12	120.78 (12)	O31—N3—C32—C31	−34.53 (17)
N1—C1—C11—C16	62.58 (14)	C31—C32—C33—C34	4.57 (18)
C21—C1—C11—C16	−59.70 (14)	N3—C32—C33—C34	−177.56 (10)
C16—C11—C12—C13	−2.0 (2)	C32—C33—C34—C35	−1.18 (18)
C1—C11—C12—C13	177.51 (12)	C32—C33—C34—N4	−178.72 (11)
C11—C12—C13—C14	0.2 (2)	O41—N4—C34—C33	4.97 (17)
C12—C13—C14—F1	179.46 (13)	O42—N4—C34—C33	−176.16 (12)
C12—C13—C14—C15	1.4 (2)	O41—N4—C34—C35	−172.63 (12)
F1—C14—C15—C16	−179.17 (13)	O42—N4—C34—C35	6.24 (18)
C13—C14—C15—C16	−1.1 (2)	C33—C34—C35—C36	−2.44 (18)
C12—C11—C16—C15	2.3 (2)	N4—C34—C35—C36	175.09 (11)
C1—C11—C16—C15	−177.19 (12)	C34—C35—C36—C31	3.01 (18)
C14—C15—C16—C11	−0.8 (2)	C34—C35—C36—N5	−176.52 (11)
N1—C1—C21—C22	−30.91 (14)	O1—C31—C36—C35	−179.08 (11)
C11—C1—C21—C22	91.36 (12)	C32—C31—C36—C35	0.01 (16)
N1—C1—C21—C26	150.90 (10)	O1—C31—C36—N5	0.44 (17)
C11—C1—C21—C26	−86.83 (12)	C32—C31—C36—N5	179.53 (10)
C26—C21—C22—C23	0.24 (18)	O51—N5—C36—C35	−34.90 (18)
C1—C21—C22—C23	−177.96 (11)	O52—N5—C36—C35	142.36 (14)
C21—C22—C23—C24	0.73 (19)	O51—N5—C36—C31	145.55 (14)
C22—C23—C24—F2	179.55 (11)	O52—N5—C36—C31	−37.19 (18)

### Hydrogen-bond geometry (Å, °)

**Table:**

*D*—H···*A*	*D*—H	H···*A*	*D*···*A*	*D*—H···*A*
N2—H721···O1^i^	0.909 (17)	1.924 (17)	2.7696 (12)	154.0 (14)
N2—H721···O52^i^	0.909 (17)	2.369 (16)	3.0018 (14)	126.7 (12)
N2—H722···O1^ii^	0.926 (16)	1.927 (16)	2.7402 (13)	145.4 (14)
N2—H722···O31^ii^	0.926 (16)	2.345 (16)	3.0791 (15)	136.0 (13)
C3—H3A···O52^i^	0.99	2.60	3.0002 (16)	104.
C13—H13···O32^iii^	0.95	2.39	3.2159 (16)	145.
C23—H23···O42^iv^	0.95	2.61	3.2827 (17)	129.

Symmetry codes: (i) *x*+1, −*y*+1/2, *z*+1/2; (ii) −*x*+1, *y*+1/2, −*z*+1/2; (iii) −*x*+1, −*y*+1, −*z*; (iv) −*x*, *y*−1/2, −*z*+1/2.
